# Role of *Cdkn2a* in the Emery–Dreifuss Muscular Dystrophy Cardiac Phenotype

**DOI:** 10.3390/biom11040538

**Published:** 2021-04-06

**Authors:** Gloria Pegoli, Marika Milan, Pierluigi Giuseppe Manti, Andrea Bianchi, Federica Lucini, Philina Santarelli, Claudia Bearzi, Roberto Rizzi, Chiara Lanzuolo

**Affiliations:** 1 Istituto Nazionale di Genetica Molecolare Romeo ed Enrica Invernizzi, 20122 Milan, Italy; pegoli@ingm.org (G.P.); milan@ingm.org (M.M.); andrea.bianchi89@libero.it (A.B.); lucini@ingm.org (F.L.); santarelli@ingm.org (P.S.); claudia.bearzi@cnr.it (C.B.); roberto.rizzi@cnr.it (R.R.); 2 Department of Oncology and Hemato-Oncology, University of Milan, 20122 Milan, Italy; pierluigi.manti@unimi.it; 3 FIRC Institute of Molecular Oncology, 20139 Milan, Italy; 4 Consiglio Nazionale delle Ricerche (CNR), Istituto di Ricerca Genetica e Biomedica, 20138 Milan, Italy; 5 Consiglio Nazionale delle Ricerche (CNR), Institute of Biomedical Technologies (ITB), 20054 Milan, Italy

**Keywords:** Emery–Dreifuss muscular dystrophy, *Cdkn2a* locus, Lamin A/C, p16^INK4a^, heart, dilated cardiomyopathy, cellular senescence

## Abstract

The *Cdkn2a* locus is one of the most studied tumor suppressor loci in the context of several cancer types. However, in the last years, its expression has also been linked to terminal differentiation and the activation of the senescence program in different cellular subtypes. Knock-out (KO) of the entire locus enhances the capability of stem cells to proliferate in some tissues and respond to severe physiological and non-physiological damages in different organs, including the heart. Emery–Dreifuss muscular dystrophy (EDMD) is characterized by severe contractures and muscle loss at the level of skeletal muscles of the elbows, ankles and neck, and by dilated cardiomyopathy. We have recently demonstrated, using the *LMNA* Δ*8–11* murine model of Emery–Dreifuss muscular dystrophy (EDMD), that dystrophic muscle stem cells prematurely express non-lineage-specific genes early on during postnatal growth, leading to rapid exhaustion of the muscle stem cell pool. Knock-out of the *Cdkn2a* locus in EDMD dystrophic mice partially restores muscle stem cell properties. In the present study, we describe the cardiac phenotype of the *LMNA* Δ*8–11* mouse model and functionally characterize the effects of KO of the *Cdkn2a* locus on heart functions and life expectancy.

## 1. Introduction

**Figure 1:**
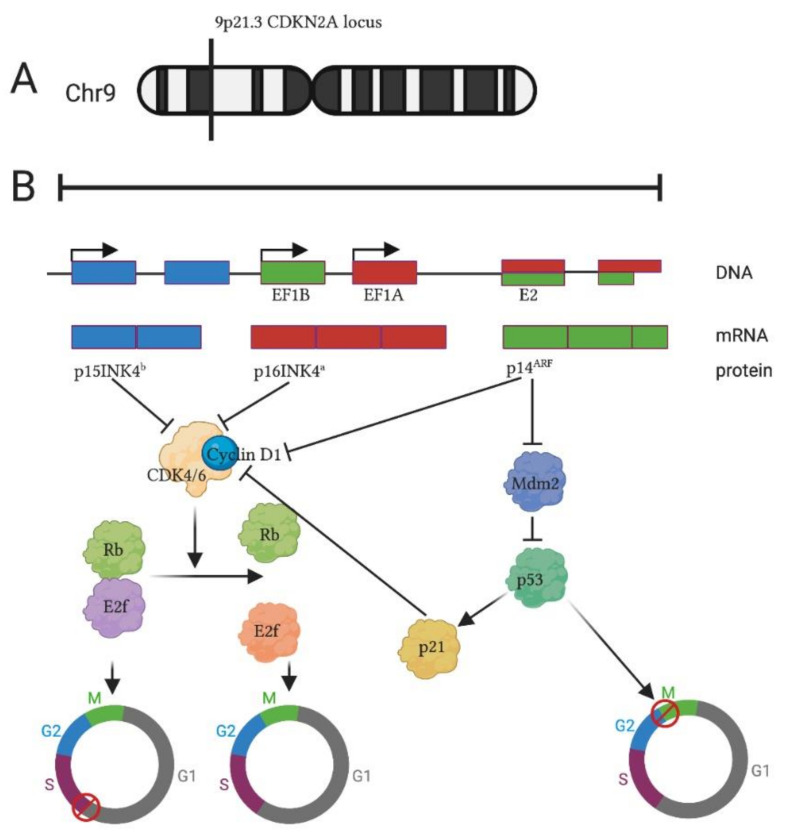
The *CDKN2A/B* loci and regulated pathways. (**A**) Localization of the locus of interest on human chromosome 9. (**B**) The products of the locus collaborate to block the cell cycle by inhibiting the retinoblastoma (RB)–E2F pathway.

The protein p16^INK4a^ is part of the INK4 family of proteins (INhibitor of Cyclin-Dependent Kinase 4) together with p15^INK4b^, p18^INK4c^ and p19^INK4d^ [1]. This group of regulators plays a crucial role in cell cycle inhibition and tumor suppression [2,3]. In humans, p16^INK4a^ is transcribed from the *Cdkn2a* locus, alternatively called INK4/ARF (ARF, Alternative Reading Frame) on chromosome 9p21.3 [1,4,5] (Figure 1). The locus contains two genes, p16^INK4a^ and ARF (the latter is named p19 in mice and p14 in humans), that, although endowed with their promoter and first exons (E1α for p16^INK4a^ and E1β for ARF), share Exons 2 and 3 (E2 and E3). E1α is spliced to E2 and E3 to produce p16^INK4a^, while E1β, upon splicing to E2 and E3, imposes a frameshift that generates p19^ARF^ (p14^ARF^ in human), a protein with different amino acid sequence. p15^INK4b^, transcribed from the flanking *CDKN2B* locus, shares 85% identity in its amino acid sequences with p16^INK4a^ [2] and both negatively regulate the pRB–E2F (retinoblastoma-Transciption Factor E2) pathway involved in cell cycle control [4] (Figure 1).

During the G1 phase, hypo-phosphorylated RB aggregates with E2F, preventing the activation of genes involved in cell proliferation [1]. In the late G1 phase, cyclin-dependent kinase 4 or 6 (CDK4/6) phosphorylates RB, provoking E2F release and S phase entry. Expression of p16^INK4a^ results in CDK4/6 inhibition and leads to cell cycle arrest at G1 phase [2,4].

ARF stops the cell cycle acting in response to aberrant cell growth or oncogenic stresses. Upon activation of several oncogenes such as Rat Sarcoma (Ras), Adenovirus early region 1A (E1A), E2F and cellular- Myelocytomatosis (c-MYC), the intracellular levels of ARF increase, causing Mouse Double Minute 2 (MDM2) inhibition and the activation of a p53-dependent transcriptional program. This molecular cascade leads to an arrest in both the G1 and G2 phase mediated by the upregulation of p21, a p53 target that is able to inhibit cyclin-dependent kinases [2,4,6,7].

*Cdkn2a* is one of the most intensively studied tumor suppressor loci, as several malignant tumors show abnormalities in the *Cdkn2a* sequence and expression status [2,3,4,5]. Specifically, in the case of p16^INK4a^, the most frequent genetic alterations are homozygous deletions, while epigenetic alterations are mediated by the Polycomb group of proteins (PcG), evolutionarily conserved epigenetic repressors that regulate higher-order chromatin structures [3,4,8,9,10,11]. Notably, even though the most common hypothesis is that the reduced expression of p16^INK4a^ is the leading cause of neoplastic progression, p16^INK4a^ overexpression has been frequently found to be associated with a poor prognosis in cancer patients [1,3,12], suggesting that p16^INK4a^ levels are tightly regulated in healthy tissues.

In parallel with the role of *Cdkn2a* in cell cycle control, p16^INK4a^ has been intensively studied for its role in cellular senescence [12] and cell differentiation [13]. Elevated levels of p16^INK4a^ induce senescence of progenitor stem cells [14,15], characterized by cell cycle blockage, DNA damage accumulation and decreased capability to remove free radicals [14,16]. Growing pieces of evidence point out the importance of p16^INK4a^ in muscle senescence, a process called sarcopenia, the age-dependent loss of muscle mass and function that can worsen the standard quality of life [17]. In later stages of mouse aging (28–32 months), subsequent to p21 expression, p16^INK4a^ expression increases and establishes the irreversible senescence program [6,18,19]. In line with recent reports showing that suppression of the *Cdkn2a* locus improves cell and tissue regeneration properties [20,21,22,23,24,25], *Cdkn2a* knock-out (KO) or knock-down (KD) [18] allow the better capacity of muscle stem cells (MuSCs) to regenerate and proliferate when occasional or chronic injuries occur in senescent skeletal muscles [18,24,26].

Senescence and Cyclin-Dependent Kinase Inhibitors (CDKI) expression in muscle stem cells are typical traits of Emery–Dreifuss muscular dystrophy (EDMD) [24,27,28], a form of muscular dystrophy affecting 1–9 in 1,000,000 patients worldwide, presenting slowly progressive muscle weakness and atrophy (ORPHA:261). The autosomal dominant form of EDMD (EDMD2) is due to heterozygous mutations in the *LMNA* gene encoding for Lamin A/C protein [29,30]. Different mechanisms have been proposed as a driving force for EDMD pathogenesis and progression [31]; among them are the epigenetic mechanisms that involve Lamin A/C and the PcG proteins, whose interplay is paving the way to understand why EDMD displays a broad spectrum of symptom variability and uncorrelated genotype–phenotype in patients [24,31,32,33,34,35].

The cardiomyopathy occurring in EDMD2 is correlated with high incidence of heart blockage and ventricular arrhythmias, resulting in a more aggressive phenotype than other inherited ones [36]. Surprisingly, heart failure without prior cardiac symptoms can be the first manifestation of the disease [37,38,39]. Dilated cardiomyopathy (DCM) [40,41,42] is interrelated with cardiac electrophysiologic defects, like sinus node dysfunction, progressive atrioventricular blockage, paroxysmal atrial fibrillation and ventricular arrhythmias [37,43,44,45,46,47]. The penetrance and expressivity of these symptoms show both inter- and intra-familial variability [48].

The cardiac phenotype of EDMD has been deeply investigated in the *Lamin^H222P/H222P^* mouse model [49,50], a model of EDMD carrying the same H222P genetic mutation found in patients. In this specific murine model, the heart dynamics associated with EDMD pathology are partially recapitulated: dilated cardiomyopathy and arrhythmias are the most evident phenotype, although no sudden deaths have been observed. Before the appearance of the severe cardiac phenotype, several signaling pathways strongly activated under stress conditions are abnormally regulated: levels of Wingless-related integration site (WNT) and β-catenin are decreased [51], while the Mitogen-Activated Protein Kinase (MAPK) proteins Extracellular signal-Regulated Kinase 1/2 (ERK1/2), p38α and Jun Nuclear Kinase (JNK) are hyperactivated [52,53,54,55,56,57,58,59], together with the a serine/threonine protein kinase- mammalian Target of Rapamycin Complex 1 (AKT-mTORC1) pathway [52]. Interestingly, by using bioinformatical tools, it has been demonstrated that even the Transforming growth factor beta (TGF-β) pathway, linked to fibrosis, is upregulated [49,52].

Molecular alterations that could affect heart functions, however, are not a prerogative of the *Lamin^H222P/H222P^* model: it has been recently reported that patient-specific induced pluripotent stem cell (iPSC)-derived cardiomyocytes (carrying mutations in the *LMNA* gene) display aberrant calcium homeostasis, leading to arrhythmias at the single-cell level that could be partially rescued by pharmacological and molecular inhibition of the Platelet-derived growth factor (PDGF) [60]. In another mouse model of DCM, tet-off bigenic mice expressing lamin (D300N) mutant protein in cardiac myocytes [61], a significant downregulation of retinoblastoma (RB) expression has been found, accompanied by transcriptional upregulation of other loci, *Cdkn2a* included, supporting the role of p16^INK4a^ in heart dysfunction [62].

Few cardiac studies have been conducted on another EDMD mouse model, the *LMNA* Δ*8*–*11*, due to the shorter life of the homozygous mutant mice that encounter premature death. While at birth, wild-type, heterozygous and homozygous *LMNA* Δ*8*–*11* mice are indistinguishable, after 2–3 weeks from birth, *LMNA* Δ*8*–*11* −/− mice show a reduction in growth and display typical traits of muscular dystrophy, dying before 8 weeks of age [63]. The cardiac phenotype of dystrophic *LMNA* Δ*8*–*11 −/−* mice at 4–6 weeks of postnatal age has been classified as DCM with limited compensatory hypertrophy [64]. Furthermore, some *LMNA* Δ*8*–*11* −/− mice show sudden death in the first 20 days of life, recapitulating all heart-associated defects reported in human EDMD. It is also known that heterozygous *LMNA* Δ*8*–*11 +/−* mice, even in the absence of skeletal myopathy, display some cardiac abnormalities late in adulthood, i.e., at approximately 50 weeks [65], which strictly recapitulate the progression of the disease in EDMD patients. Altogether, this evidence confirms that Lamin A’s absence or haploinsufficiency influences cardiac performance and can generate DCM.

In this work, we present the possible beneficial effects of genetic *Cdkn2a* locus ablation on the cardiac functions of the most severely affected EDMD mice (*LMNA* Δ*8*–*11* −/−).

## 2. Materials and Methods

### 2.1. Ethical Approval

Heterozygous *B6.129S1(Cg)-Lmnatm1Stw/BkknJ* mice (*LMNA* Δ*8*–*11* +/−) [63] and *Cdkn2a +/−* mice [66] (in which both p16^INK4a^ and p19^ARF^ were ablated) were used to obtain our model of interest. The relevant genotypes were obtained by crossing double heterozygous mice *LMNA* Δ*8*–*11 +/− Cdkn2a +/−*. Pup genotyping was performed within the first 7 days after birth on a small part of the skin, under general anesthesia. All the experimental procedures were performed under the ethical approval of the Italian Ministry of Health and the Institutional Animal Care and Use Committee (authorization No. 83/2019-PR). The animals were maintained in an authorized animal facility at San Raffaele Hospital, Milan (authorization No. N. 127/2012-A).

### 2.2. Mice Genotyping

DNA was extracted from a small amount of skin, obtained after ear tagging, using Phire tissue direct PCR master mix (Thermo Scientific, F170L, Waltham, MA, USA), then amplified using the following primers spanning the Lamin A locus: 5′-GCTTCGAGTGACTGTGACAC-3′; 5′-GTCCCCATCACTTGGTTGTC-3′; 5′-ACCGGTGGATGTGGAATGTG-3′. Primers for the *Cdkn2a* locus were previously described in Serrano et al. [66].

### 2.3. Survival and Weight Control

Mice were monitored daily and weighted starting from Day 5 after birth to death. For weight assessment, we considered 3–5 mice for each time point. After 30 days, the number of weight measurements dropped due to premature death.

### 2.4. Immunohistochemistry

Hearts were collected from anesthetized 30-day-old (P30) mice after being perfused with 50 mM KCl (Sigma, P9333, St. Louis, MO, USA), according to the experimental protocol approved by the Italian Ministry of Health. Hearts were embedded in Killik medium for inclusion (Bio-optica, 05-9801, Milan, Italy), frozen in pre-cooled isopentane (Sigma, 277258, St. Louis, MO, USA) and sectioned into pieces with 8 μm thickness (Leica CM1850 cryostat, Wetzlar, Germany). Before proceeding with the immunofluorescence protocol, samples were warmed at room temperature (RT) for 30 min and then fixed in ice-cold acetone for 10 min. After 2 washes in Phosphate-buffered saline (PBS), sections were permeabilized with 0.1% Triton x-100 (Sigma, T8787, St. Louis, MO, USA) for 10 min at RT and washed again in PBS. Samples were later blocked using 5% Bovine Serum Albumin (BSA) (Sigma, A7030, St. Louis, MO, USA) for 1 h prior to over/night (O/N) incubation at 4 °C with primary antibodies diluted in 0.5% BSA. The primary antibodies anti-cardiac troponin T (cTNNT, 1:100 dilution, Abcam, ab33589, Cambridge, MA, USA), anti-*α*-smooth muscle actin (α-SMA, 1:200 dilution, Sigma-Aldrich, A2547, St. Louis, MO, USA), anti-Connexin43 (Cx43, 1:100 dilution, Cell Signaling CST, #3512, Danvers, MA, USA), anti- Marker Of Proliferation Ki-67 (Ki67, 1:100 dilution; Abcam, ab15580, Cambridge, MA, USA) and anti-N-Cadherin (N-Cadherin, 1:800 dilution, BD, 610921) were used. The following day, the slides were washed in PBS for 10 min before the incubation with fluorescent-conjugated secondary antibodies (1:1000 dilution) for 2 h at RT. Fluorescent phalloidin, tetramethyl-rhodamine B isothiocyanate-conjugated (Sigma, P1951, St. Louis, MO, USA), was used at 50 μg/mL concentration. To quantify the number of capillaries, the sections were stained with isolectin B4 Fluorescein isothiocyanate (FITC)-conjugated antibody for 1 h at 37 °C (ISO/B4, 1:50 dilution; Sigma-Aldrich, L2140, St. Louis, MO, USA). The apoptotic index was evaluated by a Terminal deoxynucleotidyl transferase-mediated dUTP nick-end labeling (TUNEL) assay following the protocol given by the manufacturer (ApoAlert DNA Fragmentation assay kit, 630107). Cell nuclei were counterstained with 4′,6-diamidino-2-phenylindole (DAPI) (1:1000, Sigma, D9542, St. Louis, MO, USA) before mounting the slides with Prolong Glass Antifade (Thermo, P36984, Waltham, MA, USA). All the images were acquired with a confocal microscope (Leica SP5 using LAS AF software, Wetzlar, Germany) and processed using ImageJ software. The fibrotic area was assessed by Masson′s Trichrome assay (Bio-Optica, 04-010802, Milan, Italy) according to the manufacturer′s protocol. The images of all sections were acquired using a Leica optical time-lapse microscope. The fibrotic area size was expressed (in percentages) as a ratio of the fibrotic area (stained in blue) on the total area. To quantify the number of vessels, α-SMA-positive cells were divided by the total area of the section. Differently from vessels, capillary density was expressed as the number of isolectin B4-positive capillaries divided by the number of nuclei. For Connexin43 or N-Cadherin localization analysis, both signals were processed from every field with Fiji software (https://imagej.net/Fiji accessed on 23 March 2021) for background removal and transformation into a binary mask. The total area of singly positive Connexin43 or N-Cadherin and doubly positive Connexin43/N-Cadherin signals was measured and then a ratio between the co-localizing area and the total Connexin43 area was used as a quantification of Connexin43 localization on intercalated discs (IDs). For the TUNEL assay and Ki67 labeling, the positive nuclei were normalized on the total number of nuclei in the tissue field.

### 2.5. Real-Time PCR

Murine hearts were collected post mortem at Day 0 (P0) and Day 30 (P30) after birth. Total RNA was extracted from the whole heart using Tissue Ruptor (Qiagen, 9002755, Germantown, MD, USA) and TriReagent (Sigma, T9424, St. Louis, MO, USA) following the standard procedure: 1 µg of RNA from each sample was retrotranscribed into cDNA using the QuantiTect reverse transcription kit (Qiagen, 205313, Germantown, MD, USA) and amplified in the presence of 5 µL of SYBR select master mix (Thermo Fisher, 4472908, Waltham, MA, USA) using the Quant Studio 5 Real-Time PCR System (Thermo Scientific, A28140, Waltham, MA, USA). All the reactions were performed in a final volume of 10 µL and with a technical triplicate. Expression was calculated by normalizing Threshold Cycle (Ct) values on Glyceraldehyde 3-phosphate dehydrogenase (GAPDH) and relative to the average of the wild type (*LMNA* Δ*8–11 +/+ Cdkn2a +/+*) control samples. The primer sequences used for transcriptional analyses were the following: GAPDH: 5′-GTATGTCGTGGAGTCTACTGG-3′, 5′-TCGTGGTTCACACCCATCAC-3′; TGFβ: 5′-CAACCCAGGTCCTTCCTAAA-3′, 5′-GGAGAGCCCTGGATACCAAC-3′; Collagen 1a1: 5′-CCTCAGGGTATTGCTGGACA-3′, 5′-GAAGGACCTTGTTTGCCAGG-3′; Collagen 1a2: 5′-GGAACAAATGGGCTCACTGG-3′, 5′-CAAGTCCTCTGGCACCTGTA-3′; Collagen 3a1: 5′-CCCAACCCAGAGATCCCATT-3′, 5′-GGTCACCATTTCTCCCAGGA-3′; Fibronectin1: 5′-CCCCATTCCAGGACACTTCT-3′, 5′-AGGGTTCTTCATCAGTGCCA-3′; αSMA: 5′-CCTCTGGACGTACAACTGGT-3′, 5′-GGTAGTCGGTGAGATCTCGG-3′; αFAP: 5′-CACCTGATCGGCAATTTGTG-3′, 5′-CCCATTCTGAAGGTCGTAGATGT-3′; Connexin43: 5′-CAATTCCTCCTGCCGCAAT-3′, 5′-GCCCCATTCGATTTTGCTCT-3′.

### 2.6. Ecocardiography Measurements

Transthoracic echocardiography was performed on 15-day-old (P15) and 30-day-old (P30) mice using the Visual Sonic-Vevo 2100 imaging system and a MS-400 transducer, which was optimized for mice cardiovascular imaging. Before the procedure, the mice were anesthetized using 0.5–1% isoflurane according to the experimental protocol approved by the Italian Ministry of Health and kept under a hot lamp for proper thermoregulation, keeping their heartbeat at approximately 500 bpm (beat per minute). Parameters and data were obtained from M-mode recordings of at least 3 consecutive measurements along the parasternal short axis and considering the mean measures of 3 or more cardiac cycles. Data were analyzed using Vevo lab 3.2.0 software. Considering the typical pathology of EDMD, we consider the measure of fractional shortening (FS) as a significant comparison parameter that measures heart contraction, calculated as follows:

(1) $$FS=\frac{\left(LVID;d-LVID;s\right)}{LVID;d}\;\times \;100,$$

where left ventricular interior diameter during diastole is *LVID;d* and left ventricular diameter during systole is *LVID;s*. Other measurements performed are as reported in the formulae below: Equation (2): ejection fraction (EF); Equation (3): wall thickness during systole and diastole (*WT;s* and *WT;d*, respectively); Equation (4): left ventricle volume during systole and diastole (*LV Vol;s* and *LV Vol;d*, respectively); Equation (5): left ventricle mass (*LV Mass AW*).

(2) $$EF=\frac{\left(LV\;Vol;d-LV\;Vol;s\right)}{LV\;Vol;d}\;\times \;100,$$

(3) $$WT;s,d=\frac{\left(LVAW;s,d+\;LVPW;s,d\right)}{2}\;,$$

(4) $$LV\;Vol;s,d=\frac{7.0}{2.4+LVID;s,d}\;\times \;LVID;s,d^{3},$$

(5) $$LV\;Mass\;AW=\left({\left(LVID;d+LVPW;d+IVS;d\right)}^{3}-LVID;d^{3}\right)\;\times \;1.053,$$

where left ventricular anterior wall during systole or diastole is *LVAW;s,d*, left ventricular posterior wall during systole or diastole is *LVPW;s,d* and interventricular septum during diastole is *IVS;d*.

### 2.7. Statistics

All the data are represented using Graph Pad Prism 6. The sample size (*n*) for each experiment is described in the relative figure legend. All the statistical analyses were performed with parametric tests (one-way or two-way ANOVA) with Graph Pad Prism 6 (3–7 animals per group). When the datasets did not follow the normal distribution, we chose the non-parametric Kruskal–Wallis test. For Supplementary Figure S1, we evaluated the normal distribution of data with D′Agostino and Pearson, and Shapiro–Wilk tests. Statistical analysis of survival curves was performed with the Gehan–Breslow–Wilcoxon test.

### 2.8. Graphics

Figure 1 was generated by using a regularly licensed version of Biorender.

## 3. Results

### 3.1. Cdkn2a KO Ameliorates Life Span of Dystrophic LMNA Δ8–11 −/− Mice

We recently demonstrated that the absence of Lamin A in *LMNA* Δ*8–11 −/−* mice during postnatal growth causes aberrant transcriptional programs at the level of MuSCs, leading to defective identity [24,26]. We also reported that *Cdkn2a* genetic ablation restores MuSCs’ properties [24]. Here, we investigated the impact of *Cdkn2a* KO on *LMNA* Δ*8–11 −/−* heart dysfunctions.

**Figure 2:**
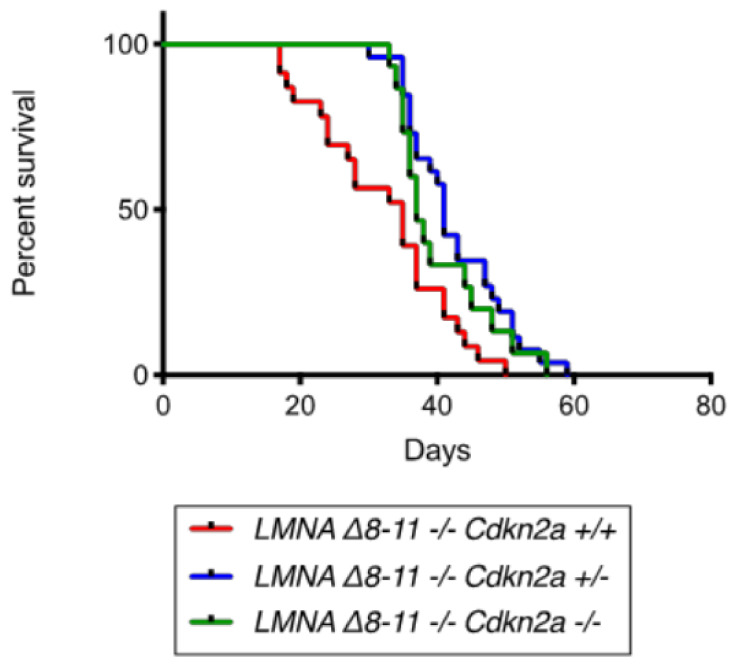
Survival curves of *LMNA* Δ*8–11 −/−* mice with different *Cdkn2a* backgrounds. Survival curves of the *LMNA* Δ*8–11 −/− Cdkn2a +/+* (**red**), *LMNA* Δ*8–11 −/− Cdkn2a +/−* (**blue**) and *LMNA* Δ*8–11 −/− Cdkn2a −/−* (**green**). *n* = 15–26. Statistical tests were performed by the Gehan–Breslow–Wilcoxon test. *p*-values: *LMNA* Δ*8–11 −/− Cdkn2a +/+* vs. *LMNA* Δ*8–11 −/− Cdkn2a +/−*; *LMNA* Δ*8–11 −/− Cdkn2a +/+* vs. *LMNA* Δ*8–11 −/− Cdkn2a* −/−.

We first monitored growth and muscle loss in mice by measuring their body weight on a daily basis (Figure S1). We found only slight differences between *LMNA* Δ*8–11 −/− Cdkn2a+/+* and *LMNA* Δ*8–11 −/− Cdkn2a−/−* genotypes during the early stages of postnatal growth. Analyzing the number of premature deaths, we noticed that the survival rate of dystrophic *LMNA* Δ*8–11 −/−* mice rapidly decreased, being 83% at Day 20 and 57% at Day 30 of postnatal growth. On the other hand, no sudden deaths could be found in the *Cdkn2a* mutated background (Figure 2) and the survival rate remained at 100% until Day 30 of postnatal growth. Altogether, these data suggest that a putative deregulation of the cell cycle during late heart development in *LMNA* Δ*8–11 −/−* mice might be at the origin of sudden death by heart failure during postnatal growth.

### 3.2. Cdkn2a KO Improves the Cardiac Function of Dystrophic LMNA Δ8–11 −/− Mice

To evaluate cardiac function in vivo, we performed trans-thoracic echocardiography. Mice were monitored at 15 days of postnatal growth, the timepoint when the skeletal muscle dystrophic symptoms started to appear, and 1 month after birth (Videos S1–S5), when mice were proximal to death as seen by the excessive muscle loss and cardiac involvement.

**Figure 3:**
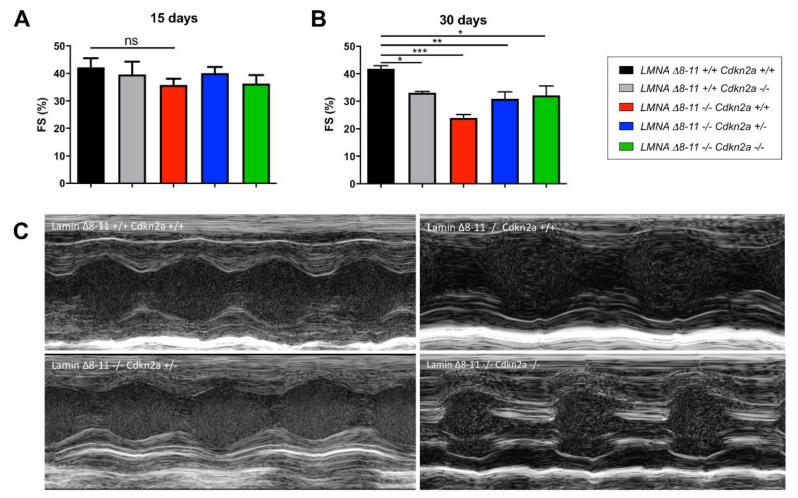
Echocardiographic results of *LMNA* Δ*8–11 −/−* mice with different *Cdkn2a* backgrounds. (**A**) Fractional shortening measures obtained at 15 days. *n* = 3–6. (**B**) Fractional shortening measures obtained at 1 month. *n* = 3–5. (**C**) Representative images of indicated genotypes of M-mode acquisitions in 1-month-old mice. Error bars represent ± Stanbdard Error of the Mean (SEM). Statistical tests were performed with one-way ANOVA with multiple comparisons. * *p* < 0.05; ** *p* < 0.01; *** *p* < 0.001. ns, not significant.

We compared the measures of fractional shortening (FS), a parameter that measures contraction performance that is widely used to assess left ventricular dysfunction (LV_dys_) (Figure 3A,B) (see Methods in Section 2). The echocardiographic analysis 15 days after birth showed slight impairment of FS in the *LMNA* Δ*8–11 −/−* background animals compared with the *LMNA* Δ*8–11 +/+* (control groups) (Figure 3A). The average value of FS found in the two control groups was 40%, while it decreased to 35% in the *LMNA* Δ*8–11 −/−* mice. The contraction deficit was also confirmed by measurement of the ejection fraction (EF) (Figure S2), a measure of the pumping efficiency of the heartbeat. At 30 days after birth, both FS and EF parameters showed a further decrease in *LMNA* Δ*8–11 −/− Cdkn2a +/+* animals, dropping to 24% (Figure 3B,C and Figure S2). On the other hand, the average value of FS was relatively compensated in *LMNA* Δ*8–11 −/− Cdkn2a +/−* and *LMNA* Δ*8–11 −/− Cdkn2a −/−* mice, resulting in a less drastic cardiac phenotype (Figure 3B,C and Figure S2). Other M-mode measurements performed on 30-day-old mice (Videos S1–S5) showed (i) a decrease in the wall thickness in systole in dystrophic *LMNA* Δ*8–11 −/− Cdkn2a +/+* mice partially recovered in *Cdkn2a* KO backgrounds (Figure S2), (ii) a reduction of the left ventricular volume in diastole and (iii) a significant drop of the Left Ventricular (LV) mass in a *LMNA* Δ*8–11 −/−* background not recovered in the absence of the *Cdkn2a* locus (Figure S2). These data are compatible with the smaller dimensions and weight of *LMNA* Δ*8–11 −/−* mice compared with the *LMNA* wild-type (wt) background. Taken together, these findings suggest the coexistence of multiple heart defects in mice lacking the *LMNA* gene. Hearts from *LMNA* Δ*8–11 −/− Cdkn2a +/−* and *LMNA* Δ*8–11 −/− Cdkn2a −/−* double mutants, despite presenting the same morphological defects as dystrophic *LMNA* Δ*8–11 −/− Cdkn2a +/+* mice, display partially restored functionality.

### 3.3. LMNA Δ8–11 −/− Mice Accumulate Fibrosis during Postnatal Heart Development

Myocardial fibrosis is the most common feature of dystrophic hearts [67] and one of the leading causes of any later cardiac pathology [68]. Considering the key *Cdkn2a* role in cell cycle control, we decided to test the progression of fibrotic infiltration by studying the transcript profile of the whole hearts of newborn mice, when cardiomyocytes are still able to replicate, and 30-day-old mice, when cardiomyocytes are entirely differentiated.

**Figure 4:**
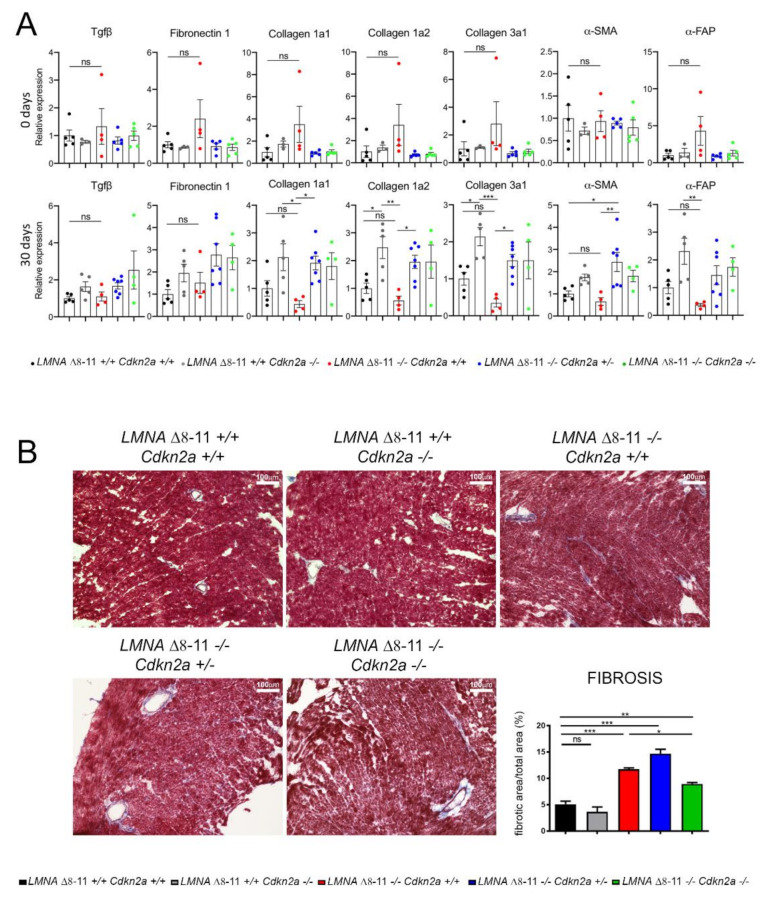
Evaluation of heart fibrosis. (**A**) Quantitative real-time analysis of fibrosis- and stress condition-related genes in the heart. The upper line represents the data obtained from newborn mice (*n* = 3–5); the lower line represents the data obtained from 1-month-old mice (*n* = 4–7). (**B**) Representative images of Masson′s trichrome staining on 1-month-old mice. The graph illustrates the fibrotic index calculated as fibrotic area/total area × 100. Scale bar represents 100 μm. Error bars represent ± SEM. Statistical tests were performed with one-way ANOVA with multiple comparisons. * *p* < 0.05; ** *p* < 0.01; *** *p* < 0.001. ns, not significant.

We tested our samples for the most common stress and fibrosis markers known in the literature: Tgfβ, a cytokine that regulates fibroblast activation during inflammation [69]; Fibronectin 1, a protein necessary for proper collagen deposition in the heart [70]; Collagen 1a1, Collagen 1a2 and Collagen 3a1, three different subunits of collagen that confer more or less elasticity on muscle tissue [71]; and α-SMA and α-FAP, both markers of fibroblast activation [72]. We highlighted a peculiar fibrosis dynamic in which *LMNA* Δ*8–11 −/− Cdkn2a +/+* mice at 0 days presented a slightly, non-significant increase in the expression of fibrotic markers (Figure 4A), which was rescued in *Cdkn2a* +/− or −/− mutated backgrounds. On the other hand, in 1-month-old animals, *LMNA* Δ*8–11 −/− Cdkn2a +/+* exhibited a trend of downregulation of all fibrotic-related genes, which was recovered in the *Cdkn2a* KO backgrounds. Unexpectedly, the histological analysis revealed a different picture with an increase in both perivascular and interstitial fibrosis in 1-month-old dystrophic *LMNA* Δ*8–11 −/− Cdkn2a +/+* mice (Figure 4B). The percentage of fibrotic area was reduced and was rescued only in the complete absence of the *Cdkn2a* locus. The accumulated fibrosis observed in 1-month-old *LMNA* Δ*8–11 −/− Cdkn2a +/+* mice could result from the initial upregulation seen at birth (Figure 4A). Alternatively, but not mutually exclusively, *LMNA* Δ*8–11 −/−* dysfunctional hearts may activate, at the transcriptional level, some compensatory repressive mechanisms to counteract the deposition of fibrosis.

### 3.4. Alteration of LMNA Δ8–11 −/− Cardiac Tissue Is Partially Recovered in a Cdkn2a KO Background

**Figure 5:**
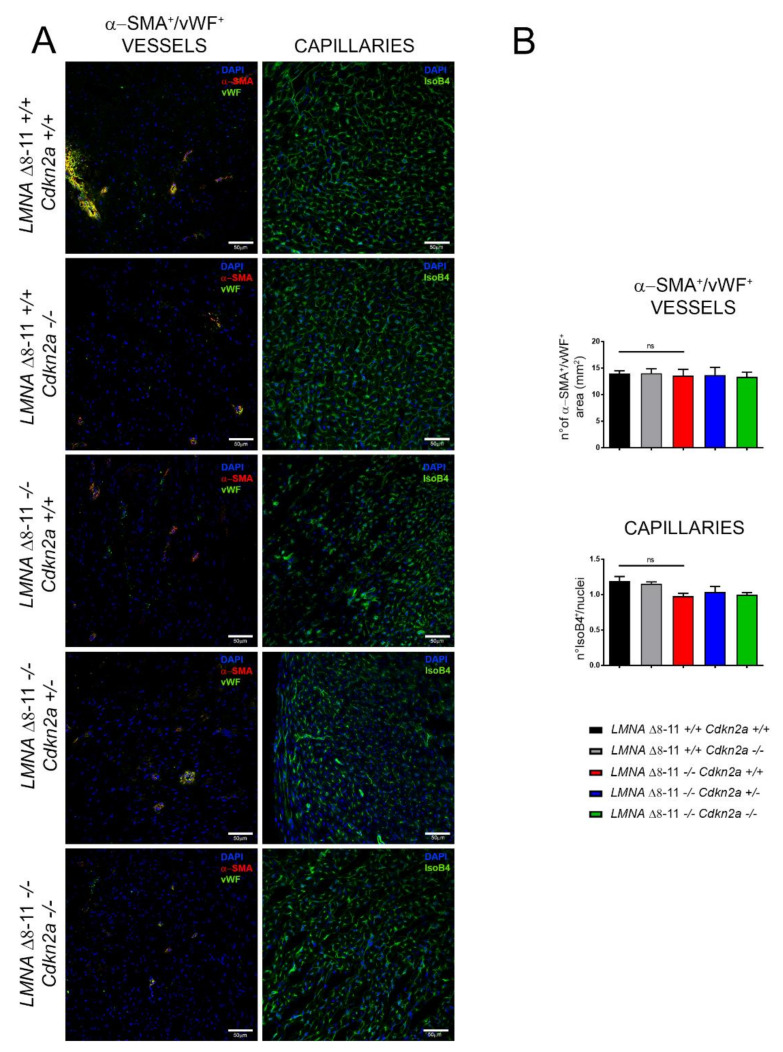
Histological analysis on heart sections. (**A**) Representative confocal images for smooth muscle actin- (α-SMA-) and von Willebrand factor- (vWF-) positive vessels (red and green; left panels) and isolectin B4-positive capillaries (green; right panels) in 1-month-old mice. Nuclei were counterstained with DAPI. The scale bar represents 50 μm. (**B**) The graphs show the ratio of α-SMA- and vWF-positive vessels on the total area (mm^2^) (upper panel)(*n* = 4–5) and capillary density as the number of isolectin B4-positive capillaries divided by the number of nuclei (lower panel)(*n* = 3–5). Error bars represent ±SEM. Statistical tests were performed with one-way ANOVA with multiple comparisons. ns, not significant.

**Figure 6:**
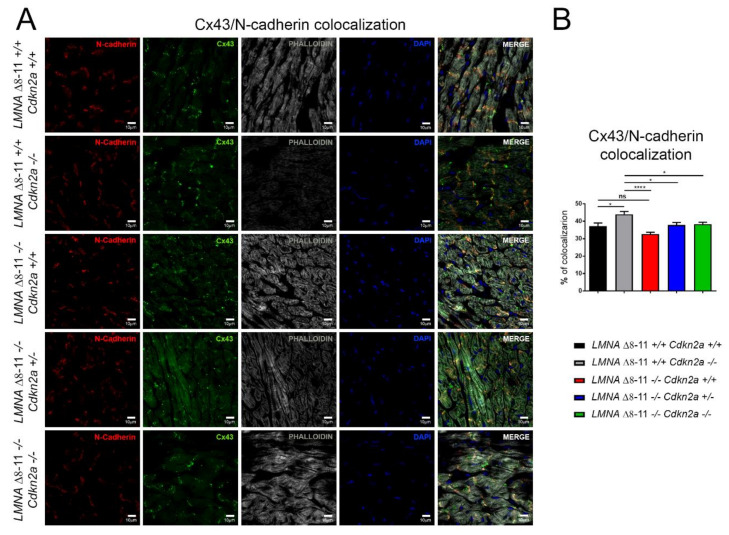
Histological analysis of heart sections on 1-month-old mice. (**A**) Representative confocal images for N-Cadherin-positive intercalated discs (IDs) (red; left panels), transmembrane gap junction Connexin43 (green; middle panels) and the F-actin cardiomyocyte cytoskeleton (White; right panels). Nuclei were counterstained with DAPI. The scale bar represents 10 μm. (**B**) Graph showing the proportion of Connexin43 localized at IDs. Error bars represent ± SEM. *n* = 4–5. Statistical analysis was performed with one-way ANOVA with multiple comparisons. * *p* < 0.05; **** *p* < 0.001. ns, not significant.

To further analyze the molecular pathways altered in *LMNA* Δ*8–11 −/−* mice and possibly recovered in the absence of *Cdkn2a*, we performed immunofluorescence staining. We first examined the number of α-SMA- (smooth muscle actin-) and vWF- (von Willebrand factor-) positive vessels and the capillary density, generally involved in the amount of oxygen and nutrients that support the repair process of the damaged tissue. We did not find significant differences between wt and *LMNA* Δ*8–11 −/−* mice (Figure 5A,B), even though we detected a slight decrease in capillary density in the dystrophic *LMNA* Δ*8–11 −/−* mice. These data corroborate previous reports showing that EDMD dystrophy is not accompanied by tissue degeneration [73]. Delocalization of CX43, with consequent alteration of heart contractile function, was observed in cardiomyopathies of multiple origin [74,75]. Thus, we decided to monitor the distribution of Connexin43 (CX43) on the fibers. In healthy hearts, this protein preferentially localizes at intercalated discs between adjacent cardiomyocytes, where it constitutes the gap junctions, essential for the propagation of action potentials and maintenance of the correct heartbeat [76]. Together with gap junctions, intercalated discs also host adheren junctions, enabling force transmission across the sarcolemma, which are easily recognizable by their principal protein component N-cadherin [77]. Although we did not find significant transcriptional differences in the CX43 gene across distinct genotypes (Figure S3A), we observed a general increase in CX43 staining in the *LMNA* Δ*8–11 −/−* mice (Figure S3B), suggesting an alteration of post-transcriptional or post-translational regulation of protein levels in the absence of Lamin A. Furthermore, we observed a slight decrease in the proportion of CX43 localizing at N-cadherin-positive intercalated discs in *LMNA* Δ*8–11 −/−* mice, which was partially recovered upon ablation of the *Cdkn2a* locus (Figure 6A,B).

**Figure 7:**
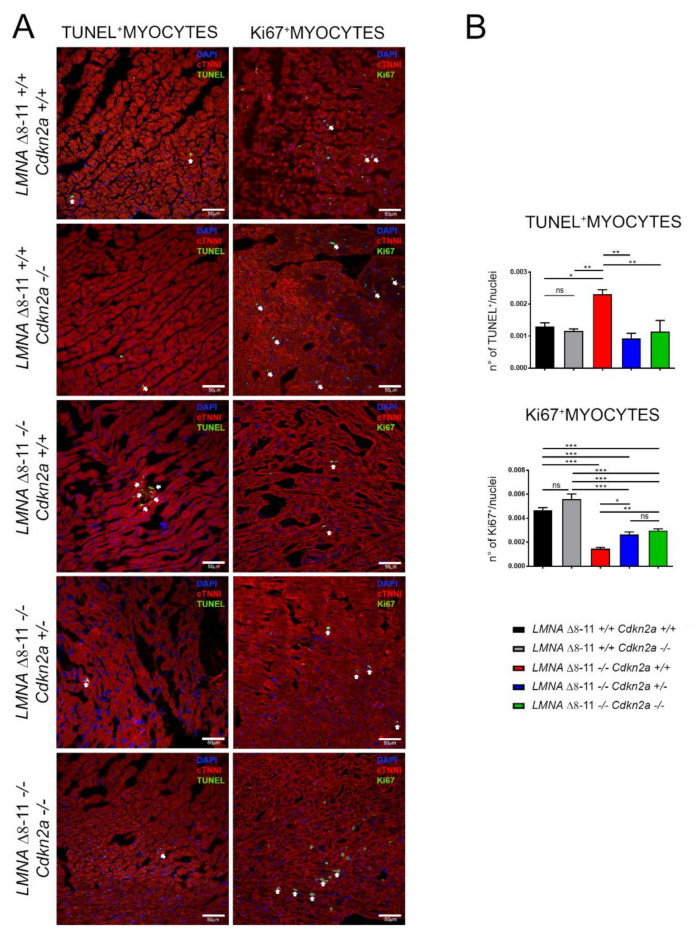
Apoptotic and proliferation assay on heart sections. (**A**) Confocal images of cTNNi-positive cardiomyocytes (red) and apoptotic (green; left panels) or proliferative nuclei stained for Ki67 (Green; right panels). Nuclei were counterstained with DAPI. The scale bar represents 50 μm. (**B**) The graphs show the ratio between TUNEL (upper panel) or Ki67-positive (lower panel) cardiomyocytes and total nuclei. Error bars represent ± SEM. *n* = 3–5. Statistical tests were performed with one-way ANOVA with multiple comparisons. * *p* < 0.05; ** *p* < 0.01; *** *p* < 0.001. ns, not significant.

Finally, we quantified the apoptotic rate of cardiomyocytes by assessing the presence of fragmented DNA with a TUNEL assay. All *LMNA* Δ*8–11 −/− Cdkn2a +/+* mice showed a significantly higher apoptotic index compared with healthy littermates, confirming a premature and aberrant blockage in the cell cycle (Figure 7A,B). The absence of one or two alleles of *Cdkn2a* is sufficient to significantly reduce the number of apoptotic cardiomyocytes (Figure 7A,B). These results were further confirmed by staining with Ki67, a marker of active proliferation. In fact, the quantification of Ki67 staining showed a substantial decrease in cardiomyocyte proliferation in dystrophic *LMNA* Δ*8–11 −/−* hearts and recovery in the *Cdkn2a* KO background (Figure 7A,B). Importantly, *LMNA* Δ*8–11+/+ Cdkn2a −/−* mice did not exhibit an increase in cell proliferation, suggesting that in a non-dystrophic condition, the lack of *Cdkn2a* function does not necessarily activate cell proliferation. Taken together, these results reveal that *Cdkn2a* plays an essential role in the regulation of cardiomyocyte fitness in the heart, and its ablation in *LMNA* Δ*8–11 −/−* dystrophic mice is enough to restore the physiological number of cardiomyocytes.

## 4. Discussion

Shortly after birth, cardiomyocytes enter a cell proliferation block. This event, together with the lack of a resident stem cell population in the heart, determines the low regeneration capability of this organ. Cardiac regenerative strategies, from the induction of cardiomyocyte proliferation to cardiac cell reprogramming and transplantation, represent a vast field of study aimed at recovering myocardial performance [78,79,80].

In recent years, several pieces of evidence have pointed out the increased capability of *Cdkn2a* KO cells to regenerate after injury [20,21,22,23]. Strikingly, these observations were extended to cardiac contractile cells, where the inactivation of p16^INK4a^ and ARF increased the capability of the whole heart in vivo and cardiomyocytes in vitro to regenerate or proliferate after ischemia, showing a functional recovery after injury, a smaller scars size and enhanced myocardial repair [21,22,81].

Emery–Dreifuss muscular dystrophy (EDMD) is a syndrome caused by Lamin A mutations. Most patients present a significant heart pathology, mainly caused by electrical conduction defects [73]. It is estimated that 10% of EDMD patients die of sudden heart failure with no prior cardiac symptoms, turning the study of asymptomatic hearth defects into an unmet clinical need [65,82]. The penetrance and expressivity of DCM-associated cardiac electrophysiological defects show both inter- and intra-familial variability, suggesting an involvement of the individual epigenetic background in the severity of the disease [44,73,83,84,85]. We recently demonstrated that Lamin A haploinsufficiency causes epigenetic transcriptional aberrancies, leading to a dysfunctional muscle stem cell niche [24,26]. As part of this work, we report that *Cdkn2a* genetic ablation alleviates Lamin A-dependent skeletal muscular dystrophy [24]. Here we show that before the 30th day of life, no sudden deaths were registered in *LMNA* Δ*8–11 −/− Cdkn2a−/−* mice (Figure 2). These data, supported by immunohistochemical analyses showing aberrant proliferation and apoptotic death in dystrophic hearts (Figure 7), suggest that a deregulated cell cycle in late heart development (both in pre- and postnatal phases) could culminate in sudden heart failure.

The heart′s capability to regenerate and substitute cardiomyocytes in adulthood after any kind of injury has long been debated, but a univocal truth has not been reached yet. Different studies have proposed several molecular mechanisms that cardiomyocytes could undertake to re-enter the replicative cell cycle but have never demonstrated heart regeneration in physiological conditions [78,79,80].

It is known that during development, there is a phase known as hyperplastic growth [86,87], when cardiomyocytes increase in number, then, during hypertrophic growth, only 0.5–2% of cardiomyocytes can replicate, while the others grow only in dimension. In mice, after the very first heartbeat at E7.5 (embryonic development Day 7.5), the precursors of cardiomyocytes actively replicate until Day E11.5 and, at 12 days after birth, stop proliferating [87] (Table 1).

The role of Lamin A during heart development has been rarely addressed, as, for a long time, it has been thought that Lamin A was expressed only after birth. Lamin A expression in prenatal heart development at E12.5 was discovered only in 2011 [88]. Thus, reasonably, *Cdkn2a* defects in Lamin dystrophy will manifest between E12.5, when *LMNA* starts to be expressed, and 12 days after birth, when cardiomyocyte proliferation stops. We believe that this time window is fundamental for physiological heart development, and lack of Lamin A, causing a premature block in the cell cycle, does not allow the complete maturation of the heart.

Although the abnormal proliferation of fibroblasts in the heart is described as pathological when associated with muscular dystrophy or myocardial infarction, reparative fibrosis is an essential early compensatory mechanism to preserve the structural heart integrity and to regulate tissue stiffness [89]. In physiological conditions, the presence of fibroblasts in the cardiac tissue is required to coordinate the electrical stimulation of cardiomyocytes [90,91,92]. Nevertheless, the excessive accumulation of interstitial fibrosis generates a barrier between cardiomyocytes, impairing normal heart electrical communication.

The role of *Cdkn2a* locus in heart myofibroblasts regulation is under debate, with findings indicating that both *Cdkn2* haploinsufficiency and overexpression can result in induction of fibrosis [93,94]. This indicates that fine-tuned regulation of the *Cdkn2a* locus is a key factor in determining the right amount of cardiac myofibroblasts. In dystrophic *LMNA* Δ*8–11 −/− Cdkn2a +/+* mice, we found an accumulation of perivascular and interstitial fibrosis accompanied by an unexpected transcriptional downregulation of fibrosis-related pathways (Figure 4), suggesting the presence of counteracting mechanisms participating in fibrosis activation.

## 5. Conclusions

According to the World Health Organization (WHO), cardiovascular diseases are the leading cause of death globally. The discovery of new molecular and epigenetic mechanisms at the basis of different cardiac pathologies may give an advantage to the development of new therapies. Even if related to a rare disease such as EDMD, any further information on dilated cardiomyopathy may be eventually useful for new promising treatments for diseases with similar progression. Although further studies will be needed to translate these results into clinical practice, our findings, revealing in dystrophic *LMNA* Δ*8–11 −/−* mice the role of the cell cycle in the postnatal heart development might contribute to the identification of new pathways that could be used for the classification of individual risk for sudden cardiac death.

## Figures and Tables

**Table 1:** Key characteristics of human and mouse hearts.

	Human	Mouse
First heartbeat	3 weeks	E7.5
Four chambers visible	1.5 months	E9.5
Decline in proliferation	2 months	E11.5
Lamin A/C expression	Not addressed	E12.5
Stop proliferation	1 week after birth	12 days after birth

## Supplementary Materials

The following are available online at https://www.mdpi.com/2218-273X/11/4/538/s1, Figure S1: Body weight in dystrophic mice with different *Cdkn2a* backgrounds. Graphical representation of body weight (g) during post-natal growth of the different genotypes. Number of measurements: *LMNA*Δ*8–11* −/− *Cdkn2a+/+ = *32; *LMNA*Δ*8–11* −/− *Cdkn2a+/-* = 39; *LMNA*Δ*8–11* −/− *Cdkn2a*−/− *=* 32. Number of analysed mice: *LMNA*Δ*8–11* −/− *Cdkn2a+/+ =* 12; *LMNA*Δ*8–11* −/− *Cdkn2a+/-* = 11; *LMNA*Δ*8–11* −/− *Cdkn2a*−/− *=* 8. Nonparametric statistical test performed with Kruskal-Wallis test. P value of *LMNA* Δ*8–11* −/− *Cdkn2a +/+* vs *LMNA* Δ*8–11* −/− *Cdkn2a* −/− = 0.0464. The dataset are represented fitting the curves with no linear regression using the second order (quadratic) polynomial equation. Figure S2. Echocardiographic measurements on 15-days- and 30-days-old mice. Ejection fraction, wall thickness (WT) in systole (s) and diastole (d), left ventricular mass (AW) and left ventricular volume in systole (s) and diastole (d) are shown. In the upper line are represented 15-days mice, n = 3–6, in the lower line are represented 30-days. Error bars represent ± SEM, n = 3–5. Statistical test performed with one-way ANOVA with multiple comparisons. * *P* < 0.05; ** *P* < 0.01; *** *P* < 0.001. ns, not significant. Figure S3. (A) Quantitative real-time amplifications for Connexin43 marker in heart at 0-days and 1 month, n = 3–7. (B) Graph showing proportion of areas positive for Connexin-43 only (green), N-cadherin only (red) or both (yellow) over the total Connexin43 and/or N-cadherin positive area in the different genotypes. n = 4–5. Error bars represent ± SEM. Statistical test performed with two-way ANOVA with multiple comparisons. * *P* < 0.05; ** *P* < 0.01; *** *P* < 0.001. ns, not significant. Video S1. Echocardiographic recording (M-mode) of a representative *Lamin* Δ*8–11 +/+ Cdkn2a +/+* mouse at 30 days. Video S2. Echocardiographic recording (M-mode) of a representative *Lamin* Δ*8–11 +/+ Cdkn2a* −/− mouse at 30 days. Video S3. Echocardiographic recording (M-mode) of a representative *Lamin* Δ*8–11* −/− *Cdkn2a +/+* mouse at 30 days. Video S4. Echocardiographic recording (M-mode) of a representative *Lamin* Δ*8–11* −/− *Cdkn2a +/-* mouse at 30 days. Video S5. Echocardiographic recording (M-mode) of a representative *Lamin* Δ*8–11* −/− *Cdkn2a* −/− mouse at 30 days.
