# Supplementary material for: Role of Cdkn2a in the Emery–Dreifuss Muscular Dystrophy Cardiac Phenotype
Source: Biomolecules. 2021 Apr 6;11(4):538. doi: 10.3390/biom11040538 (PMC8103514; doi:10.3390/biom11040538)
Supplement: Supplementary file 1 [file biomolecules-11-00538-s001.zip › biomolecules-11-00538-s001.pdf]

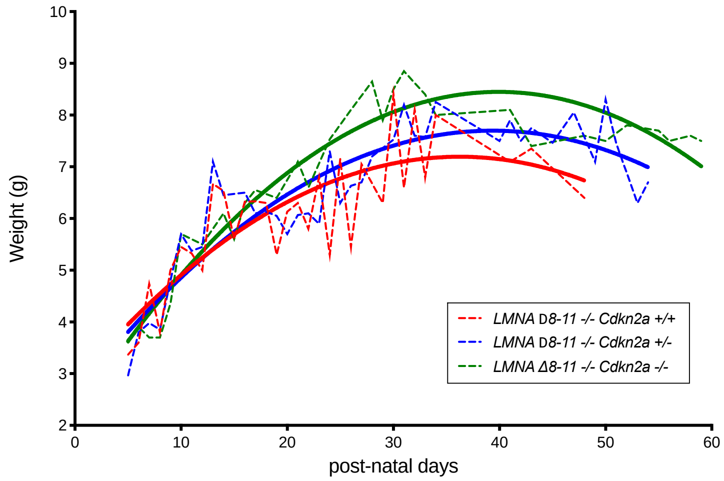

Figure S1. Body weight in dystrophic mice with different *Cdkn2a* backgrounds. Graphical representation of body weight (g) during post-natal growth of the different genotypes. Number of measurements: *LMNAΔ8-11 -/- Cdkn2a*+/+=32; *LMNAΔ8-11 -/- Cdkn2a*+/-=39; *LMNAΔ8-11 -/- Cdkn2a*-/-=32. Number of analysed mice: *LMNAΔ8-11 -/- Cdkn2a*+/+=12; *LMNAΔ8-11 -/- Cdkn2a*+/-=11; *LMNAΔ8-11 -/- Cdkn2a*-/-=8. Nonparametric statistical test performed with Kruskal-Wallis test. P value of *LMNA Δ8-11 -/- Cdkn2a* +/+ vs *LMNA Δ8-11 -/- Cdkn2a -/-* = 0.0464. The dataset are represented fitting the curves with no linear regression using the second order (quadratic) polynomial equation.

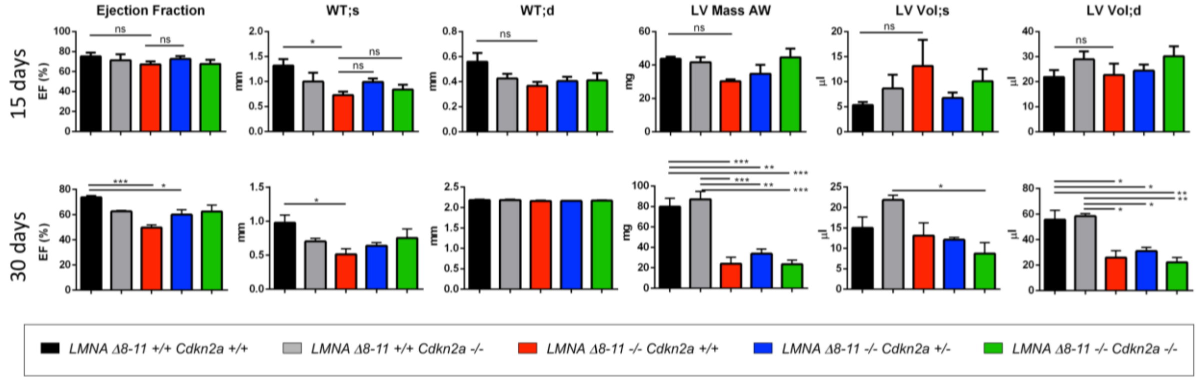

Figure S2. Echocardiographic measurements on 15-days- and 30-days-old mice. Ejection fraction, wall thickness (WT) in systole (s) and diastole (d), left ventricular mass (AW) and left ventricular volume in systole (s) and diastole (d) are shown. In the upper line are represented 15-days mice, n=3-6, in the lower line are represented 30-days. Error bars represent ±SEM, n=3-5. Statistical test performed with one-way ANOVA with multiple comparisons. **P* < 0.05; ***P* < 0.01; ****P* < 0.001. ns, not significant.

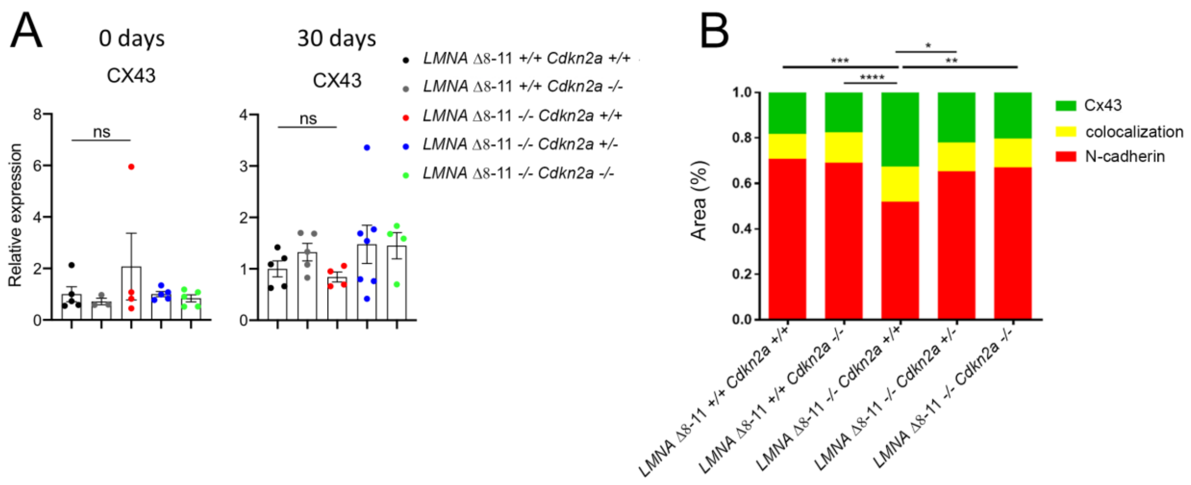

Figure S3. (A) Quantitative real-time amplifications for Connexin43 marker in heart at 0-days and 1 month, n=3-7. (B) Graph showing proportion of areas positive for Connexin-43 only (green), N-cadherin only (red) or both (yellow) over the total Connexin43 and/or N-cadherin positive area in the different genotypes. n=4-5. Error bars represent ±SEM. Statistical test performed with two-way ANOVA with multiple comparisons. *P < 0.05; **P < 0.01; ***P < 0.001. ns, not significant.

Video S1. Echocardiographic recording (M-mode) of a representative *Lamin Δ8-11* +/+ *Cdkn2a* +/+ mouse at 30 days.

Video S2. Echocardiographic recording (M-mode) of a representative *Lamin Δ8-11* +/+ *Cdkn2a -/-* mouse at 30 days.

Video S3. Echocardiographic recording (M-mode) of a representative *Lamin Δ8-11 -/- Cdkn2a* +/+ mouse at 30 days.

Video S4. Echocardiographic recording (M-mode) of a representative *Lamin Δ8-11 -/- Cdkn2a* +/- mouse at 30 days.

Video S5. Echocardiographic recording (M-mode) of a representative *Lamin Δ8-11 -/- Cdkn2a -/-* mouse at 30 days.
